# Roles of Autophagy Induced by Natural Compounds in Prostate Cancer

**DOI:** 10.1155/2015/121826

**Published:** 2015-03-03

**Authors:** V. Naponelli, A. Modernelli, S. Bettuzzi, F. Rizzi

**Affiliations:** ^1^Department of Biomedicine, Biotechnology and Translational Research, University of Parma, Via Volturno 39/a, 43125 Parma, Italy; ^2^Centre for Molecular and Translational Oncology (COMT), University of Parma, Parco Area delle Scienze 11/a, 43124 Parma, Italy; ^3^National Institute of Biostructure and Biosystems (INBB), Viale Medaglie d'Oro 305, 00136 Rome, Italy

## Abstract

Autophagy is a homeostatic mechanism through which intracellular organelles and proteins are degraded and recycled in response to increased metabolic demand or stress. Autophagy dysfunction is often associated with many diseases, including cancer. Because of its role in tumorigenesis, autophagy can represent a new therapeutic target for cancer treatment. Prostate cancer (PCa) is one of the most common cancers in aged men. The evidence on alterations of autophagy related genes and/or protein levels in PCa cells suggests a potential implication of autophagy in PCa onset and progression. The use of natural compounds, characterized by low toxicity to normal tissue associated with specific anticancer effects at physiological levels *in vivo*, is receiving increasing attention for prevention and/or treatment of PCa. Understanding the mechanism of action of these compounds could be crucial for the development of new therapeutic or chemopreventive options. In this review we focus on the current evidence showing the capacity of natural compounds to exert their action through autophagy modulation in PCa cells.

## 1. Introduction

In Europe, prostate cancer (PCa) is the first most frequent diagnosed malignancy and the third-leading cause of cancer death in men [1]. Although patients with an early androgen-dependent and localized tumor have a good prognosis, the survival rate decreases notably when the tumor eventually becomes androgen-independent and progresses to a hormone-refractory disease leading to metastasis formation. At present, patients with hormone-sensitive PCa at early-stage can be treated with surgery, radiotherapy, and/or hormonal therapy (i.e., surgical or medical castration). Nevertheless, the disease can progress into castration-resistant and metastatic PCa, for which the only treatment option is chemotherapy with docetaxel. Therefore, further investigations are required to elucidate the mechanisms underlying onset and progression of PCa and to develop new strategies for therapy and prevention. Increasing evidence supports a key role of autophagy in cancer development, drawing researchers' attention because of its potential implication as a drug target in anticancer treatments.

## 2. Autophagy and PCa

### 2.1. The Autophagic Machinery and Its Regulation

In eukaryotic cells, proteins are degraded through two major proteolysis systems: the proteasome degradation and autophagy. The ubiquitin-proteasome system is the major catabolic pathway for short-lived proteins, while autophagy is a process through which long-lived proteins, damaged organelles, and other waste intracellular material are delivered to lysosomes for degradation. Autophagy is constitutively active at low levels in order to preserve cellular homeostasis but strongly induced by stressful conditions, such as nutrient deprivation, growth factor depletion, oxidative stress, hypoxia, irradiation, and anticancer drug treatments. Under these stressful conditions, autophagy is believed to act primarily as a first protective response. Nevertheless, autophagy may also participate in cell death, constituting an alternative caspase-independent cell death mechanism called type II (or macroautophagy-related) programmed cell death [2, 3]. The importance of autophagy in physiology and pathophysiology is underlined by the finding of an association of autophagic dysfunctions with the development of important diseases including neurodegenerative disorders, myophaties, and cancer [4].

**Figure 1:**
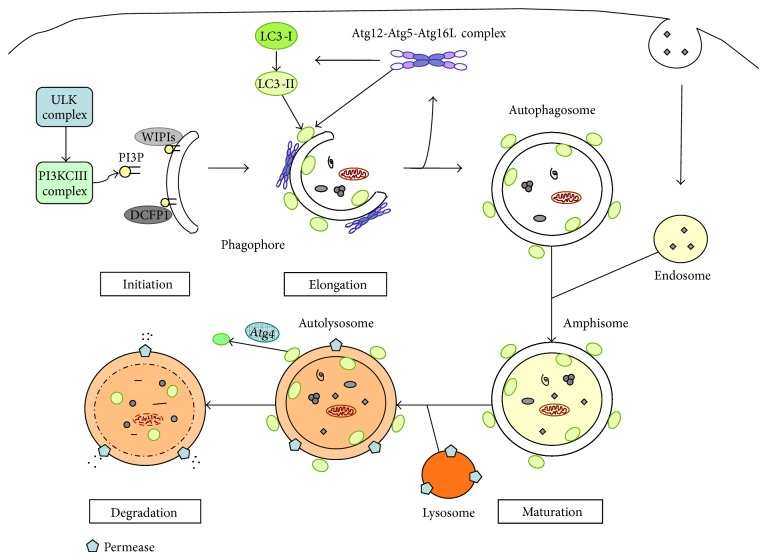
Schematic representation of autophagy. The process begins with the nucleation of the phagophore, followed by its elongation and expansion and its closure to form the double-membrane autophagosome. The autophagosome maturates first through fusion with endosome, producing an amphisome, and then with lysosome to form the final autolysosome, where the inner membrane and the sequestered content are degraded by the lysosomal hydrolases. Finally, the resulting macromolecules are returned to cytoplasm by permeases for reuse. In the figure, the core molecular machinery of autophagy is also illustrated, including the ULK complex that is required for autophagy induction, the PI3KCIII complex and the PI3P interacting proteins, such as WIPIs and DCFP1, which contribute to the phagophore formation and elongation. Also the LC3-II and Atg12-Atg5-Atg16L complexes take part to the elongation step. The Atg12-Atg5-Atg16L complex resides on the outer membrane of the phagophore and dissociates from the completed autophagosome. The LC3-II complex is present on both sides of the phagophore and autophagosome, but it is released by Atg4-mediated deconjugation from the outer membrane after autophagosome maturation.

Autophagy is an evolutionarily conserved dynamic cellular catabolic process. Many autophagy-related (Atg) proteins take part in the various steps of the autophagic pathway. So far more than 36 Atg genes have been characterized in yeast and the majority of them have orthologues in mammals. Many mammal-specific proteins with multiple functions in autophagy have also been identified [5]. When autophagy is induced, Atg proteins associate following a hierarchical order (Figure 1); in mammals the first step is the formation of a preautophagosomal structure which seems to localize on endoplasmic reticulum (ER), where the uncoordinated-51-like kinase (ULK) and the class III phosphatidylinositol 3 kinase (PI3KCIII) complexes are early recruited to start the double-membrane structure nucleation [6]. The ULK complex is composed of ULK1/2 (homologs of yeast protein kinase Atg1), Atg13L, Atg101, and FIP200. Once activated, it recruits other Atg proteins and interacts with Atg9L1 and the PI3KCIII complex. Atg9L1 is a trans-membrane protein that cycles between the trans-Golgi network and the endosomes, and during starvation it localizes on autophagosomes, regulating the autophagosome size [7]. The PI3KCIII complex consists of Beclin 1, vacuolar protein sorting 15 (Vps15) and class III PI3K (Vps34). The PI3KCIII complex, through the activation of the Vps34 enzymatic activity, enriches the double-layer structure of membranes with phosphatidylinositol 3-phosphate (PI3P), which is essential for vesicle nucleation and recruiting of PI3P-interacting Atg proteins, such as Double-FYVE-containing protein 1 (DFCP1) and WD-repeat protein interacting with phophoinositides (WIPIs) (both homologues of yeast Atg18). Afterwards, during the later steps of autophagy, two ubiquitin-like protein conjugation systems participate in the elongation and maturation of autophagosome: the Atg12-Atg5-Atg16L complex and the microtubule-associated protein 1 light chain 3 (LC3)-phophatidylethanolamine (PE) complex. Atg7 and Atg10 catalyze the conjugation between Atg12 and Atg5. Then Atg12-Atg5 complex interacts noncovalently with Atg16L forming a tetrameric structure through the homooligomerization of Atg16L [5]. This final multimeric complex localizes on the outer membrane of the autophagosome and is released from the membrane just before or after the completion of autophagosomes. The second ubiquitin-like molecule is LC3 (the mammalian Atg8 homologue), which is first hydrolyzed by Atg4 to LC3-I (cytosolic form). After that, Atg7 and Atg3 mediate the conjugation of LC3-I with PE producing the lipidated form LC3-II. The Atg12-Atg5-Atg16L complex cooperates facilitating the conjugation of LC3-I with PE. LC3-II displays an apparently symmetrical distribution on both sides of the phagophore membrane [5]. The LC3-II molecules residing on the cytoplasmic side of the autophagosome are delipidated by Atg4 in order to be recycled, while the LC3-II located inside the autophagosome is degraded after fusion with the lysosome. The autophagosome maturation continues with the fusion of endosomes to form amphisomes; at last, the fusion with lysosomes generates autolysosomes, which will degrade the entrapped content.

**Figure 2:**
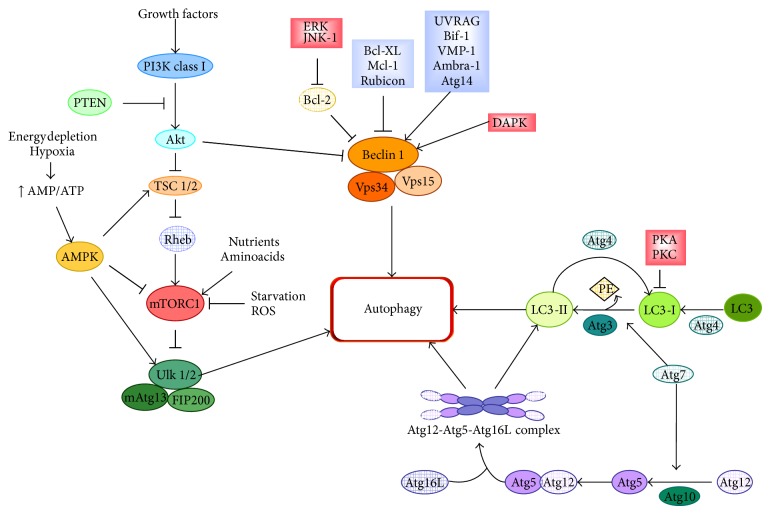
Schematic representation of the molecular regulation of autophagy. Growth factor signalling activates the PI3K/Akt/mTORC1 pathway resulting in autophagy inhibition. mTORC1 is also activated by amino acids and nutrient rich conditions, whereas starvation and oxidative stress induce autophagy via mTORC1 inhibition. Starvation and hypoxia can also induce autophagy through AMPK activation. Beclin 1-Vps34-Vps15 complex (or PI3KCIII complex) is required for the induction of autophagy, and the interaction between its components is regulated by interacting proteins (blue boxes): Rubicon, Mcl-1, and Bcl-XL/Bcl-2 are negative regulators, whereas proteins, such as UVRAG, Atg14, Bif-1, VMP-1, and Ambra-1, through their interaction with Beclin-1 and Vps34, promote the activity of the PI3KCIII complex inducing autophagy. Numerous kinases (red boxes) are involved in autophagy regulation: ERK and JNK-1 can induce autophagy by releasing Bcl-2 inhibition through its phosphorylation; Akt inhibits autophagy via Beclin 1 phosphorylation, whereas DAPK-mediated phosphorylation of Beclin 1 promotes autophagy. Finally, PKA and PKC negatively regulate autophagy acting on LC3. Atg4, Atg3, Atg7 and Atg10 are autophagy-related proteins which mediate the formation of the LC3-II complex and the Atg12-Atg5-Atg16L complex, and they may represent additional control points in the autophagic pathway.

Several extracellular (e.g., nutrient status, hormonal and therapeutic treatment) and intracellular (e.g., metabolic stressors and accumulation of misfolded proteins) stimuli are able to activate autophagy and many signaling pathways are involved in the regulation of the autophagic process (Figure 2). The mammalian target of rapamycin (mTOR) pathway is the most studied pathway regulating autophagy. The mTOR pathway involves two functional complexes: the mTOR complex 1 (mTORC1) that is an important controller of cell growth and proliferation and plays a major role in controlling autophagy, and the mTOR complex 2 (mTORC2) that is not directly implicated in autophagy modulation. The mTORC1 pathway is a key sensor of nutrient and energy status and is regulated by signals such as growth factors, amino acids and stressors. Mainly under nutrient-rich conditions, mTORC1 directly interacts with and phosphorylates ULK1 negatively affecting the ULK complex formation. Conversely, starvation inhibits mTORC1 leading to dephosphorylation-dependent activation of the ULK complex, which then translocates from the cytosol to the phagophore [8]. In addition to mTORC1, AMP-activated protein kinase (AMPK), another cell key energy sensor, can play a major role in transmitting autophagic signaling. AMPK is activated by the increase in cellular AMP/ATP ratio occurring during nutrient deprivation or hypoxia, and positively regulates the ULK complex both by direct phosphorylation of ULK1 and inhibition of mTORC1 via a pathway involving tuberous sclerosis complex 1 and 2 (TSC1/2) [8]. The PI3KCIII complex is another major point of regulation of autophagy induction. The association of Beclin 1 to the other subunits of the PI3KCIII complex is a key event for the induction of PI3P synthesis by Vps34. Beclin 1-Vps34 connection is regulated by the interaction with Bcl-2, Bcl-XL, Mcl-1 and Rubicon, which act as inhibitors, and with Atg14, UV radiation resistance associated gene (UVRAG), Bax-interacting factor-1 (Bif-1), vacuole membrane protein 1 (VMP1) and Ambra-1, which behave as activators [9, 10]. The dynamic interaction between Beclin 1 and its binding proteins is further regulated by post-translational modifications. For instance, the phosphorylation of Beclin 1 by the death associated protein kinase (DAPK) triggers the dissociation of Beclin 1-Bcl-XL/Bcl-2 complex, allowing Beclin 1 to interact with Vps34 [11], while Beclin 1 phophorylation by Akt inhibits autophagy [12]. Moreover, the phosphorylation of Bcl-2 by c-Jun N-terminal kinase 1 (JNK1) or extracellular signal-regulated kinase (ERK) reduces Beclin 1-Bcl-2 interaction leading to autophagy activation [13]. Downstream the ULK and the PI3KCIII complexes, LC3 can be down-regulated via phosphorylation by protein kinase A (PKA) or protein kinase C (PKC) [14, 15].

### 2.2. The Role of Autophagy in PCa

The role of autophagy in cancer is controversial and still not completely clarified: it has been described as a double-edged sword because of its involvement in both cell survival and tumor suppression, depending on cell type, genetic context, stage of tumor development and nature of the stressor [16, 17]. As mentioned above autophagy is an evolutionarily conserved process that allows cells to respond to changed environmental conditions preserving cellular homeostasis. This function is particularly important for cancer cells that are characterized by high metabolic demand. As a prosurvival mechanism, autophagy may be used by transformed cells to adapt to the tumor microenvironment, which is hypoxic, nutrient limiting and metabolically stressful due to the inadequate blood supply [18]. According to this concept, autophagy is mostly evident in cancer cells localized in the inner, poorly vascularized tumor regions especially during the late stage of carcinogenesis. Cancer cells residing in these tumor regions are generally cells resistant to anticancer treatments [19, 20]. Consistently, in some cancer cells, antineoplastic therapies induce autophagy as a resistance and prosurvival mechanism and, in these cases, genetic or pharmacologic autophagy inhibition can be used to increase the efficacy of the anticancer treatments [21, 22]. The protective role of autophagy has also been evidenced by studies showing an increased activation of programmed cell death pathways when Atg genes are knocked down [23].

Paradoxically, autophagy defects have been found in many human tumors: monoallelic loss of the essential autophagy gene Beclin 1 and decreased levels of the protein have been frequently found in human breast, ovarian, and prostate cancers [24]. In addition molecular analyses of tumors in Beclin 1 heterozygous mice confirmed that Beclin 1 is a haploinsufficient tumor suppressor [25, 26]. Many other Atg genes, such as Atg4, Atg5, Atg7, UVRAG, Bif-1 [27–30], and autophagy regulators, including p53, phosphatase and tensin homolog (PTEN), DAPK [31, 32], have been implicated in tumorigenesis and are considered tumor suppressors. Furthermore, many signaling molecules of the PI3K/Akt/mTOR pathway, a negative regulator of autophagy, have oncogenic properties and the constitutive activation of this pathway is very common in human tumors [22]. The loss of autophagic functions can result in accumulation of protein aggregates and damaged organelles, above all damaged mitochondria, and consequently in reactive oxygen species (ROS) production, which then promotes genome instability furthering oncogenic transformation and cancer progression [28, 33]. This evidence is indicative of the anticancer role of autophagy.

With regard to PCa, studies have indicated that autophagy is compromised in PCa cells: PTEN, the suppressor of the PI3K/Akt/mTOR pathway, is the most frequently deleted tumor suppressor gene in PCa and the PI3K/Akt/mTOR pathway is upregulated in 30–50% of PCa tumors and associated with increasing tumor stage, grade and risk of recurrence [34]. Moreover, a number of Atg genes, such as Beclin 1 and LC3 genes, map to chromosomal loci that are frequently monoallelically deleted in PCa cells [35, 36] and the protein expression of Beclin 1 ad LC3 have been demonstrated to be lower in prostate adenocarcinoma than in prostate benign hyperplasia [37]. Nevertheless, a recent study has demonstrated that about 35% of PCa shows an over-expression of key autophagy proteins (LC3 and p62) directly related to a high Gleason score, indicating that autophagy signaling may be important for cell survival in high-grade PCa [38].

The response of cancer cells to the autophagic stimulus can trigger cell death or cell survival depending on the integration of complex signaling pathways not yet completely clarified. Due to this two-faced role, a better understanding of the regulation and modulation of the autophagic pathway might provide new insights into cancer treatment and prevention. If the prosurvival role of cancer cell autophagy is generally accepted, intense investigations are needed to understand whether the autophagy-associated cancer cell death, induced by some drugs and natural compounds, may be exploited as a promising strategy for cancer therapy.

## 3. Natural Compounds Inducing Autophagy in PCa

Natural products are receiving increasing attention for the prevention and/or treatment of cancer because of their promising efficacy and low toxicity to normal tissue. Therefore there is a great interest in identifying new natural products active against PCa and in understanding the mechanisms of action of these compounds to exploit their properties in the development of new therapeutic or preventive treatments. Since autophagy may become a new therapeutic target for PCa treatment, in this section we will report the evidence on natural compounds able to modulate autophagy influencing PCa cell fate (Table 1).

### 3.1. Isothiocyanates

Isothiocyanates are a family of compounds derived from the myrosinase-mediated hydrolysis of glucosinolates contained in cruciferous vegetables. High intake of cruciferous vegetables may be associated with reduced risk of aggressive PCa [61, 62], and isothiocyanates are believed to be responsible for the anticancer effects of these vegetables [63]. Sulphoraphane [1-isothiocyanato-4-(methylsulfinyl)-butane] (SFN), the most studied isothiocyanate, was firstly identified as a chemopreventive agent able to both inhibit Phase 2 detoxification enzymes and induce Phase 1 enzymes involved in carcinogen activation [64, 65]. SFN, as well as other naturally occurring isothiocyanates, can also block cancer development by causing cell cycle arrest and apoptosis induction in cancer cells. SFN, benzyl isothiocyanate (BITC), phenethyl isothiocyanate (PEITC) and allyl isothiocyanate are all isothiocyanates having antitumor effects on PCa both* in vitro* and* in vivo*, without affecting normal prostate epithelial cells [66–70]; moreover, most of these isothiocyanates are able to induce autophagy in PCa cells as well as in breast, colon, and pancreatic cancer models [39–45, 71–74].

In PCa cell lines, SFN inhibited cell proliferation by causing G2-M phase cell cycle arrest and caspase-dependent apoptosis [75–78]. Orally administration of this isothiocyanate reduced prostate tumor growth and pulmonary metastasis in transgenic adenocarcinoma of mouse prostate (TRAMP) mice without causing any side effects [67] and retarded the growth of PC-3 xenografts in nude mice [78, 79]. Recently, SFN-induced autophagy in PCa cells has been reported both* in vitro* and* in vivo*. Human PCa cells (LNCaP and PC-3) treated with SFN at a dose of 40 *μ*M exhibited the hallmarks of autophagy including the formation of AVOs, the processing and the punctuate localization of LC3 [39]. Autophagy occurred before the onset of apoptosis, and presumably the sequestration of mitochondria by autophagosomes was the cause of a delayed release of cytochrome *c* and activation of intrinsic caspase cascade. This evidence, showing autophagy as a protective mechanism against apoptosis induced by SFN, was confirmed by pharmacologic inhibition of autophagy using 10 mM 3-methyladenine (3-MA), which augmented SFN-induced apoptotic cell death [39]. SFN (20 *μ*M) caused ROS generation due to the inhibition of mitochondrial respiratory chain enzymes, and these mitochondria-derived ROS initiated the apoptotic cell death and the earlier protective autophagic response in PCa cell lines [41, 80]. Autophagy induction by SFN was also observed* in vivo*: in TRAMP models, a 5-week cotreatment with SFN (1 mg by oral intubation 3 times per week) and the autophagy inhibitor chloroquine (1.2 mg by intraperitoneal injection 3 times per week) resulted in a reduction of poorly differentiated prostate tumors and lymph node metastasis compared to the untreated control group and the group treated with SFN alone. In addition, the TUNEL-positive apoptotic bodies were significantly increased by the combination of SFN with chloroquine [40]. This evidence confirms* in vivo* the cytoprotective function of SFN-induced autophagy in PCa.

Another isothiocyanate able to induce autophagy in human cancer cells is BITC [42, 71]. In PC-3 cells, BITC induced Bcl-XL phosphorylation, cell cycle arrest and subsequent apoptosis [68], and in DU145 human PCa cells, BITC was shown to induce ROS production triggering the activation of the apoptotic pathway [81]. Though in these studies BITC-mediated autophagy induction was not investigated, both Bcl-XL phosphorylation and ROS production may stimulate autophagy [10, 33]. A recent study specifically examined BITC ability to induce autophagy in human hormone-sensitive (22Rv1) and -refractory (PC-3) PCa cell lines. BITC was shown to inhibit mTOR signaling triggering autophagy in a dose- and time-dependent manner [42]. Combination of 20 *μ*M BITC and 1 mM 3-MA significantly increased BITC-induced apoptotic cell death in either 22Rv1 or PC-3 cells, showing that BITC-induced autophagy represented an early protective response, as also observed for SFN-treated PCa cells [39, 42].

PEITC is another naturally occurring isothiocyanate that has received increasing attention due to its cancer chemopreventive effects.* In vitro*, PEITC suppressed growth of PCa cells (PC-3, LNCaP and DU145) through induction of G2-M phase cell cycle arrest and apoptotis [44, 82–86]. Moreover, PEITC oral administration retarded the growth of PCa xenografts in nude mice, reduced the incidence of poorly differentiated tumors and increased the TUNEL-positive apoptotic bodies in PC-3 xenografts and TRAMP mice [44, 45, 83, 84]. Hallmarks of autophagy have been characterized* in vitro* and* in vivo*: PCa cells (LNCaP and PC-3), but not normal prostate epithelial cell line (PrEC), treated with 2.5–5 *μ*M PEITC exhibited significant accumulation of AVOs and enhanced processing and punctuate localization of LC3 [43, 44, 87]; increased expression and cleavage of LC3 were also revealed in tumor sections from mice with PC-3 xenografts gavaged with 9 *μ*M PEITC and from TRAMP mice fed 3 *μ*mol PEITC/g diet [44, 45]. Both PEITC-induced autophagy and apoptosis in LNCaP and PC-3 cell lines were strongly dependent on Atg5 protein level [44], thus proving an interrelation between the two pathways activated by PEITC treatment. PEITC, as well as other isothiocyanates, induced mitochondria-derived oxidative stress in LNCaP and PC-3 cells, and the generated ROS played a critical role in the initiation of apoptosis by induction of Bax mitochondrial translocation and cytosolic release of cytochrome *c* [43]. Nevertheless, differently from SFN and BITC, PEITC induced an autophagic process that was only partially dependent upon ROS production [43]. The treatment with 5 *μ*M PEITC resulted in the suppression of Akt/mTOR. However, overexpression of positive regulators of mTOR, Akt or Rheb, conferred only a partial protection against PEITC-mediated autophagy [44], suggesting the potential involvement of other mechanisms in the activation of the autophagic response evoked by PEITC. Remarkably, cotreatment with the autophagy inhibitor 3-MA (4 mM) or knockdown of Atg5 protein attenuated the apoptotic DNA fragmentation and the activation of caspase 3, thus suggesting that PEITC-mediated autophagy contributed to the promotion of apoptotic and nonapoptotic cell death [44].

### 3.2. Polyphenols

Polyphenols constitute one of the largest and ubiquitous group of phytochemicals: flavonoids and phenolic acids represent the most common ones in food. Epidemiological evidence suggests lower PCa risk in populations with higher consumption of major polyphenols [88, 89]. Several naturally occurring polyphenols, including resveratrol, green tea catechins and curcumin, are currently being studied for their potential role in PCa prevention and treatment. These compounds can induce both apoptotic and autophagic cell death in various type of cancers [90].

Resveratrol (3,5,40-trihydroxystilbene) is a natural nonflavonoid polyphenolic compound present in grape skin, red wine and nuts. After Jang et al. reported for the first time in 1997 the ability of resveratrol to inhibit the carcinogenic process at multiple stages, including initiation, promotion and progression [91], subsequent studies have focused on its potential chemopreventive function in many different animal models of carcinogenesis [92]. Resveratrol has been reported to have antiproliferative and proapoptotic effects on PCa cell lines [93]. Relatively few* in vivo* studies however have investigated and confirmed the effects of resveratrol on PCa prevention and treatment [92]. Nevertheless, there is no evidence from human clinical trials for resveratrol as an effective supplement for prevention and treatment of prostate diseases [94]. Resveratrol ability to induce autophagy in different cancer cell lines as either a prosurvival or a prodeath mechanism [90, 95, 96]. With regard to PCa, Li et al. showed that, in DU145 cells, resveratrol (50 *μ*M for 24 h) induced a significant increase in autophagy leading to nonapoptotic programmed cell death. Conversely, androgen-responsive LNCaP and androgen-independent C42B cells resulted relatively resistant to resveratrol treatment. The data suggested that SIRT1, a NAD-dependent histone deacetylase belonging to the family of sirtuins, could act as a positive regulator of autophagy in DU145 cells triggering the dephosphorylation of S6K, one of the key effectors of mTOR [46]. Consistently with the* in vitro* findings, treatment of 4- or 5-week-old PTEN knockout mice with resveratrol for 14 weeks was associated with reduction in the prostatic levels of mTORC1 activity and increased expression of SIRT1, supporting that the SIRT1/S6K pathway could play an important role in autophagy induced by resveratrol in PCa [46].

Green tea catechins have antitumoral and chemopreventive properties demonstrated by* in vitro* and* in vivo* studies [97–99]. Of all the catechins found in green tea, (-)-epigallocatechin-3-gallate (EGCG) is the most abundant and biologically active. In PCa cells green tea catechins, and especially EGCG, are able to modulate a plethora of cell signaling pathways crucial for cancer cell transformation and survival [97, 100–102]. Chemopreventive and chemotherapeutic effects of these polyphenols have been observed in preclinical models of PCa, including both genetic and xenograft models [103–105]. In addition, there have been 5 intervention studies evaluating the effect of green tea intake on the change in risk markers of PCa: among them, there was only one randomized, double-blind, placebo-controlled trial demonstrating the efficacy of green tea supplementation on PCa incidence [106–111]. Green tea catechins on cancer. The regulation of autophagy by EGCG seems to be dependent on concentration, cell types and stress conditions [112]. Our data reported that two prostate epithelial cell lines, PNT1a and PC-3, mimicking initial and advanced stages of PCa respectively, responded differently to the treatment with Polyphenon E, a standardized green tea extract. The treatment of PNT1a cells with 35 *μ*g/mL Polyphenon E for 24 h triggered the activation of caspases committing cells to anoikis, while 145 *μ*g/mL Polyphenon E caused PC-3 cell death through a caspase-independent necroptotic event. Autophagy was transiently activated only in PNT1a cells between 6 and 12 h of treatment as a survival response to overcome Polyphenon E-induced ER stress [47].

Curcumin is a polyphenolic compound isolated from the rhizomes of* Curcuma longa*, exhibiting anti-inflammatory, anticancer and antioxidant activities based on its chemical features and its ability to interact with multiple signaling molecules [113]. Curcumin exerts a cytotoxic and cytostatic action in many transformed cells, prevents carcinogen-induced cancer in rodents and inhibits the growth of human tumors in xenograft or orthotransplanted animal models, either as single treatment or in combination with chemotherapeutic drugs or radiation [114]. Curcumin and its derivatives have been described to inhibit different signaling pathways in cancer resulting in apoptotsis [115, 116] or in caspase-independent cell death mechanisms, like autophagy [117–120]. Curcumin-induced autophagy is generally described as a prodeath signal [119, 121, 122], however it has recently been demonstrated to exert a prosurvival and prodifferentiation role in tumor initiating cells [123] and to precede or accompany a senescence/quiescence-promoting effect in cancer cells [124–126]. Curcumin affected cell proliferation of androgen-sensitive (22Rv1), but not of androgen-independent (DU145 and PC-3) PCa cells, through the induction of G2 cell cycle arrest and modulation of Wingless (Wnt/*β*-catenin) signaling pathway. The reduction of cell viability observed after curcumin treatment (20 *μ*M for 24 h) in 22Rv1 cells was linked to autophagy induction as demonstrated by the appearance of LC3-II form and the decrease of Bcl-XL expression [48]. Bcl-XL is an antiapoptotic protein, but also an antiautophagic protein via its inhibitory interaction with Beclin 1 [9, 10]. This highlights the complex interrelationship existing between autophagy and the apoptotic cell death pathway.

Gossypol is a natural polyphenolic compound isolated from cottonseeds that acts as a BH3-mimetic small molecule pan-inhibitor of antiapoptotic Bcl-2 family members including Bcl-2, Bcl-XL and Mcl-1 [127]. Treatment with gossypol led to inhibition of cell viability and induction of apoptosis in different kinds of PCa cells and significantly inhibited angiogenesis and PCa xenografts growth [128, 129]. Unfortunately, only limited efficacy was proved in clinical trials [130, 131]. Gossypol has been reported to induce Beclin 1-dependent or -independent autophagy, with a prosurvival or a prodeath effect depending on the cancer cell type [127]. Lian et al. investigated* in vitro* and* in vivo* the mechanism leading to gossypol-induced cell death in human PCa cells expressing different levels of Bcl-2. Gossypol (10 *μ*M) preferentially induced apoptosis in PCa cells with low Bcl-2 (LNCaP, DU145 and C4-2B), whereas an autophagic cell death was observed in apoptosis-resistant, androgen independent cells with high Bcl-2 (PC-3 and CL-1) [49]. Functioning as a pan-Bcl-2 inhibitor, gossypol down-regulated Bcl-2, Bcl-XL and Mcl-1. Thus, gossypol triggered autophagy mainly via inhibition of the interaction between Beclin 1 and Bcl-2/Bcl-XL [49, 50].* In vivo* evidence confirmed that gossypol inhibited CL-1 and PC-3 xenografts tumor growth by autophagy induction [49]. Also apogossypolone, a semi-synthesized derivative of gossypol, at a concentration of 10 mg/L, was able to provoke an early activation of the autophagic pathway in both PC-3 and LNCaP cells. However, in this case, autophagy acted as a protective response against apoptosis induction [51].

### 3.3. Vitamins

In recent years, various reports have shown that vitamins, such as vitamin C and vitamin K, exhibit antioncogenic effects [132, 133]. In various cancer cell lines, autophagy has been evidenced to be evoked as a response to vitamin K or ascorbic acid treatment [52, 53, 134–140]. Autophagy triggered by vitamins has mainly been described as an alternative caspase-independent cell death pathway that supports apoptosis [134–138]. Nevertheless, autophagy has also been characterized as a prosurvival response against apoptosis in human hepatoma cells treated with vitamin K3 (a synthetic version of vitamin K) [139] and in glioblastoma cells treated with ascorbic acid [140]. In PCa, autophagy induced by vitamins can have both a prodeath and a prosurvival function depending on doses and treatment conditions [52, 53].

The treatment of different types of PCa cell lines, including androgen-dependent (LNCaP), androgen-sensitive (22Rv1) and androgen-independent cells (PC-3 and C4-2), with ascorbate (0–20 mM) for 2 h demonstrated that ascorbic acid, at concentrations clinically achievable with pharmacological intravenous infusion, could induce H_2_O_2_-dependent cytotoxicity [52, 141]. Increased conversion of LC3-I to LC3-II, punctuated pattern of GFP-LC3 signal and transmission electron microscope observations of autophagosome structures were demonstrated after exposure of PC-3 cells to 5 mM ascorbate for 6 h, evidencing the activation of the autophagic pathway [52]. Inhibition of ascorbate-induced autophagy by 3-MA treatment increased cell viability, and knockdown of Bif-1, a positive mediator of autophagy, resulted in PC-3 cell resistance to ascorbate-induced cell death, thus suggesting a prodeath role for autophagy [52].

Vitamin K3 (menadione, 2-methyl-1,4-naphthoquinone) is a synthetic derivative of vitamin K1 that has been demonstrated to exhibit anticancer activity in human cancer cell lines and to potentiate the cytotoxic effects of several chemotherapeutic agents [132]. The combination of vitamin K3 and vitamin C has shown synergistic antitumour activity against PCa* in vitro* [142–144] and* in vivo* [145, 146]. In PC-3 cells the combination of subtoxic concentrations of vitamin K3 (3 *μ*M) and ascorbic acid (0.4 mM) caused autophagy activation, which acted as a protective mechanism induced by oxidative stress [53]. In this conditions, autophagy could be overcome by the coadministration of subtoxic doses of the redox-silent vitamin E analogue *α*-tocopheryl succinate (30 *μ*M), which acted as a ROS scavenger [53]. The triple combination treatment (vitamin K3, ascorbic acid and *α*-tocopheryl succinate) was associated with synergistic/additive cytotoxic effects on PC-3 cell line and PC-3 xenografts in nude mice [53, 144], supporting that the inclusion of *α*-tocopheryl succinate in the combinatorial treatment could result in the overcoming of the prosurvival responses to ascorbic acid/vitamin K3 treatment.

### 3.4. Emerging Natural Compounds Able to Induce Autophagy in PCa

Since autophagy is involved in carcinogenesis, the ability of newly discovered or confirmed anticancer natural compounds to modulate this cellular pathway in PCa cells is receiving increasing attention, as supported by recent evidence.

Rottlerin is a natural plant polyphenol, isolated from* Mallotus philippinensis* (Euphorbiaceae), with demonstrated anticancer activity: this active compound is able to affect several cell pathways involved in survival, apoptosis, autophagy and invasion [147]. Recent data reported that in human PCa stem cells, rottlerin (0.5–2 *μ*M) induced autophagy in a dose-dependent manner by activating AMPK pathway, inhibiting the PI3K/Akt/mTOR pathway and decreasing Bcl-2 and Bcl-XL protein levels [54]. Rottlerin-induced autophagy could be characterized as a prodeath pathway linked to apoptotic cell death. In effect, the administration of autophagy inhibitors (3-MA, chloroquine or bafilomicyn A1) caused the suppression of both autophagy and apoptosis in PCa stem cells treated with rottlerin [54].

Piperine and piperlongumine, two alkaloids present in black (*Piper nigrum* Linn) and long (*Piper longum* Linn) peppers, have been recently reported to mediate antitumoral effects on human PCa cells* in vitro* and* in vivo* [55, 56, 148–150], and autophagy was one of the mechanism triggered by this active compounds [56, 151]. In particular, piperlongumine (10 *μ*M) was shown to induce autophagy in PC-3 cells by down-regulating the Akt/mTOR signaling pathway. In this case, autophagy was ROS-dependent, as cotreatment with the antioxidant N-Acetyl-L-Cysteine reversed piperlongumine effects [56]. Concomitant treatment with piperlongumine and chloroquine enhanced cell death in PC-3 cell lines and reduced growth of xenograft tumors in immunodeficient mice, demonstrating a prosurvival function of piperlongumine-mediated autophagy [56].

Ursolic acid is a natural pentacyclic triterpenoid isolated from plants and medicinal herbs and has many biological functions, including antitumor activities on PCa cells [152, 153]. In PC-3 cells, the treatment with 40 *μ*M ursolic acid for 24 h caused an early activation of autophagy via the disruption of the PI3K/Akt/mTOR pathway [57]. Ursolic acid-induced autophagy represented an early protective mechanism to allow cell escape from apoptosis [57]. Autophagy induced by ursolic acid has also been evidenced in human breast, colorectal and cervical cancer with conflicting effects on cell survival [154–156].

Marchantin M, a macrocyclic bisbibenzyl extracted from* Asterella angusta*, has anti-inflammatory and cytotoxic effects on PCa cells [58, 157, 158]. Marchantin M-triggered proteasome inhibition and ER stress, as well as suppression of the PI3K/Akt/mTOR pathway, contributed to autophagy induction in PC-3 cells [58]. Autophagy was shown to be implicated in marchantin M-mediated cell death, as demonstrated by the almost complete restoration of PC-3 cell viability after the combined treatment with pan-caspase and autophagy inhibitors [58].

Red yeast rice, produced by the fermentation of rice with fungus of the* Monascus* species, is a traditional Asian food spice that has also medicinal uses due to the anti-inflammatory, antioxidative and antitumor properties of its metabolites [159, 160]. One of these metabolites is monascuspiloin, which is able to inhibit the growth of hormone-sensitive (LNCaP) and hormone-insensitive (PC-3) PCa cells. Monascuspiloin (50 *μ*M for 12 h) induced apoptosis in both PCa cells, but preferentially in LNCaP cells, whereas the induction of the autophagic pathway via AMPK activation prevailed in PC-3 cells [59]. Autophagy induced by monascuspiloin represented a prodeath mechanism that sustained apoptosis [59]. Hallmarks of autophagy, including high expression of LC3-II, Atg5, Atg12 and Beclin 1, were also confirmed in PC-3 xenograft tumors in nude mice treated with 40–120 mg/kg monascuspiloin [59]. Furthermore, monascuspiloin was shown to sensitize PC-3 cells and PC-3 xenografts to ionizing radiation through inducing ER stress and autophagy [60].

## 4. Conclusion

Autophagy has a controversial and quite complicated role in PCa tumorigenesis. It can act as a tumor suppressor during the early stages of carcinogenesis, but it can also be used by transformed cells as a survival mechanism to overcome the stresses imposed during tumor growth. The prosurvival role of autophagy is responsible, at least in part, for the adaptive response of PCa cells to various anticancer therapies, including radiation therapy and conventional DNA damaging chemotherapy. The prodeath function of autophagy, at the moment poorly characterized, could be attributed to two separate functions: the proapoptosis function of autophagy and the induction of autophagic cell death, without the involvement of apoptosis machinery.

On one hand, the synergism between autophagy inhibitors and conventional chemotherapeutic drugs is attracting more and more attention since this strategy could overcome resistance, indeed increasing and maximizing the clinical effectiveness of PCa therapy. On the other hand, the induction of autophagic cell death could represent a promising strategy to trigger an alternative type of programmed cell death in cancer cells that have acquired resistance to apoptosis.

Many published studies, especially those based on a morphology-based definition of autophagic cell death, fail to establish the causative role of autophagy in the cell death process. It is urgently required a joint effort of many researchers to understand whether and when autophagy is a real independent cell killer and an accomplice of apoptosis, or a passive bystander effect that occurs concomitantly with cell death.

The studies summarized in this review suggest that many natural compounds induced autophagy by specifically downregulating the Akt/mTOR pathway, thus indicating that autophagy may induce cell death through a specific molecular commitment. It is noteworthy that the Akt/mTOR pathway, frequently upregulated in PCa, contributes to the disease development and progression also through an extensive crosstalk with many other signaling pathways involved in cell survival, apoptosis, growth and differentiation. Since mTOR activity can be directly or indirectly modulated by a number of upstream signaling pathways, it is mandatory to uncover the mechanisms through which these natural compounds inhibit the Akt/mTOR pathway and impact on the cell fate.

In addition, a better understanding of the molecular effectors that interconnect autophagy to programmed cell death is urgently required to look at many natural compounds as a “sustainable” hope for therapeutic anticancer strategy. There is a strong need of well designed clinical studies to answer to the question whether natural substances may have a relevant role in PCa therapeutic managing. Developing methods and techniques useful to monitor the role of autophagy* in vivo* will be fundamental to reach the target.

## Figures and Tables

**Table 1:** Functional status of autophagy induced by different natural compounds.

	*In vitro*/*in vivo* system	Dose	Mechanism	Effects on cell fate	Reference
Sulphoraphane	LNCaP and PC-3 cell lines; TRAMP mice	20–40 *μ*M; 1 mg	Mitochondria-derived ROS	Prosurvival	[39–41]
Benzyl isothiocyanate	22Rv1 and PC-3 cell lines	20 *μ*M	mTOR	Prosurvival	[42]
Phenethyl isothiocyanate	LNCaP and PC-3 cell lines; PC-3 xenograft models; TRAMP mice	2.5–5 *μ*M; 9 *μ*mol; 3 *μ*mol/g	ROS production, Akt/mTOR	Prodeath	[43–45]
Resveratrol	DU145 cell line	50 *μ*M	SIRT1/S6K/mTOR	Prodeath	[46]
Polyphenon E	PNT1a cell line	35 *μ*g/mL		Prosurvival	[47]
Curcumin	22Rv1 cell line	20 *μ*M		Prodeath	[48]
Gossypol	CL-1 and PC-3 cell lines and PC-3 xenograft models	10 *μ*M	Bcl-2-Beclin 1	Prodeath	[49, 50]
Apogossypolone	LNCaP and PC-3 cell lines	10 mg/L		Prosurvival	[51]
Ascorbate	PC-3 cell line	5 mM	ROS production	Prodeath	[52]
Vitamin K3/vitamin C	PC-3 cell line	3 *μ*M vit. K3 + 0.4 mM vit. C	ROS production	Prosurvival	[53]
Rottlerin	Human PCa stem cells	0.5–1-2 *μ*M	AMPK, PI3K/Akt/mTOR, Bcl-2-Beclin 1	Prodeath	[54]
Piperine	LNCaP and PC-3 cell lines	160 *μ*M			[55]
Piperlongumine	PC-3 cell line	10 *μ*M	ROS production, Akt/mTOR	Prosurvival	[56]
Ursolic acid	PC-3 cell line	40 *μ*M	Akt/mTOR	Prosurvival	[57]
Marchantin M	PC-3 cell line	10 *μ*M	ER stress, PI3K/Akt/mTOR pathway	Prodeath	[58]
Monascuspiloin	PC-3 cell line; PC-3 xenograft models	50 *μ*M; 40–120 mg/kg	AMPK	Prodeath	[59, 60]
